# Can a Toy Encourage Lower Calorie Meal Bundle Selection in Children? A Field Experiment on the Reinforcing Effects of Toys on Food Choice

**DOI:** 10.1371/journal.pone.0169638

**Published:** 2017-01-13

**Authors:** Martin Reimann, Kristen Lane

**Affiliations:** University of Arizona, Eller College of Management, Department of Marketing, Tucson, Arizona, United States of America; TNO, NETHERLANDS

## Abstract

The goal of this research was to test whether including an inexpensive nonfood item (toy) with a smaller-sized meal bundle (420 calories), but not with the regular-sized meal bundle version (580 calories), would incentivize children to choose the smaller-sized meal bundle, even among children with overweight and obesity. Logistic regression was used to evaluate the effect in a between-subjects field experiment of a toy on smaller-sized meal choice (here, a binary choice between a smaller-sized or regular-sized meal bundles). A random sample of 109 elementary school children from two schools in the Tucson, Arizona metropolitan area (55 females; *M*_*age*_ = 8.53 years, *SD*_*age*_ = 2.14; *M*_*BMI*_ = 18.30, *SD*_*BMI*_ = 4.42) participated. Children’s height and weight were measured and body-mass-index (BMI) was calculated, adjusting for age and sex. In our sample, 21 children were considered to be either overweight or obese. Logistic regression was used to evaluate the effect of a toy on smaller-sized meal choice. Results revealed that the inclusion of a toy with a smaller-sized meal, but not with the regular-sized version, predicted smaller-sized meal choice (*P* < .001), suggesting that children can be incentivized to choose less food when such is paired with a toy. BMI neither moderated nor nullified the effect of toy on smaller-sized meal choice (*P* = .125), suggesting that children with overweight and obesity can also be incentivized to choose less. This article is the first to suggest that fast-food restaurant chains may well utilize toys to motivate children to choose smaller-sized meal bundles. Our findings may be relevant for consumers, health advocates, policy makers, and marketers who would benefit from a strategy that presents healthier, but still desirable, meal bundle options.

## Introduction

One-third of children and adolescents now visit and consume fast food meals every day [1]. In addition, fast food consumption has increased to make up about twelve percent of daily calories for children and adolescents [2]. Moreover, fast food portion sizes have also significantly grown over the decades [3], and especially children’s fast food meals have been subject to scrutiny for having high energy density [4, 5]. In part a result of these trends, percentages of overweight and obese children in the U.S. have risen to more than one third of the population [6]. In response to child overweight and obesity, researchers have started investigating food marketing tactics that may have contributed to the pandemic. For example, toy premiums in children’s meals (e.g., McDonald’s^™^ Happy Meals and Burger King’s^™^ King Jr. Meal) have been criticized for motivating children to visit fast food restaurants [7]. Indeed, critics of food marketing suggested that, for children, toys are a key driver of fast food meal choice [8]. Relatedly, two recent systematic reviews found that character endorsers (e.g., McDonald’s Ronald McDonald) considerably increase children’s liking and preference for *energy-dense* foods [9, 10]. The effects of food marketing tactics on children, when used in this way, are especially worrisome because they likely establish and reinforce long-lasting food choice patterns [11]. Both the popularity of these tactics among food marketers and meal bundle’s link to overweight and obesity have generated suspicions that these tactics have incentivized over-consumption. In that sense, both fast food portion sizes *and* food marketing tactics are culprits in childhood overweight and obesity [5, 7]. Herein, we asked, can restaurants and food manufacturers use food marketing tactics for the better—for example, can we use toy premiums to stimulate smaller-sized portion choice?

In the present research, we were curious to know whether toy premiums could be used to incentivize children to choose a smaller-sized meal bundle (also referred to as children’s combination meals). On first sight, cutting calories out of a meal may seem like an *ineffective* strategy in motivating children’s food choice. Clearly, smaller-sized food portions are less psychologically valuable, less attention-grabbing [12], and less desirable [13] than larger-sized ones and are, therefore, often rejected by children [14]. Because a larger-sized meal portion provides more psychological “bang-for-your-buck” than a smaller-sized meal portion [12], we wondered if the lower value of a smaller-sized meal (versus a larger-sized meal) could be recuperated with psychological value gained from offering a toy premium? We conducted a field experiment to answer this question. Specifically, we tested whether including a toy with a smaller-sized children’s meal bundle (420 calories), but not with a regular-sized children’s meal bundle (580 calories), a reduction of 160 calories between the two bundles, would predict smaller-sized meal bundle choice. Alternatively, an argument could be made that toys do not alter caloric intake and would, therefore, be ineffective in altering meal-size selection [13]. However, for reasons discussed next, we are the first to argue that toys can be used as effective substitutes in place of larger portions in meal bundles.

One might expect that substitutes need to satisfy *common* physiological needs. For example, both water and juice, but not solid food, satisfy thirst and are thereby substitutable [14]. Indeed, the classic economic notion of substitutability claims two commodities are substitutable only if a decrease in the consumption of one commodity (e.g., French fries) is accompanied by a similar and opposite increase in the consumption of the other commodity (e.g., fried onion rings). The law by which this rationale follows is called the matching law [15]. However, the matching law does not consider qualitatively different stimuli. Following this logic, food (e.g., French fries) and toys, which are clearly in two different categories, are not substitutable.

Yet, we build on recent research in arguing that—less obviously so—even highly different stimuli such as food and toys can share a common psychological basis, hence allowing their behavioral substitution. Given our ancestral past, humans have learned to associate foods with appetitive and survival values, which makes food a natural reward [16, 17]. Because children can be reinforced for the receipt of toys [18], toys can thus represent artificial rewards. Theoretically, the translation of both food and toy into a common reinforcement value should facilitate choice substitution of having more food with toy. Indirect, preliminary evidence for the notion of a common physiological basis of natural and artificial rewards comes, for example, from a recent adult study that observed automatic responses (e.g., salivation) for attractive material goods to be similar to those expected for delicious foods [19]. Following this novel notion of choice substitution, we argue that toys can be substituted for larger portions of food and thereby used as positive reinforcers. Because of such common reinforcement value for food and toy, we argue that when paired with a smaller-sized meal bundle the reinforcing prowess of the toy can counterbalance the lower reinforcement value of a smaller-sized meal bundle. This value substitution will cause the smaller-sized meal bundle to be psychologically valuable, attention-grabbing, and desirable, incentivizing children to choose a smaller-sized, instead of a regular-sized, meal bundle. Left by their parents to make meal choices on their own [20], and little influenced by advertising of lower calorie meals [21], children are likely to select meal options with more calories. Our method seeks to intervene at the counter to help children make better choices.

We also questioned if there would be a difference in meal-size choice between children with higher and lower body mass index (BMI). We asked, are food cues more attention-grabbing in children with overweight and obesity and could, therefore, override the effect of the toy for children with higher BMI? This quandary seems reasonable, considering recent research showing a visual attention bias favoring food cue images among obese adults [22], decreased inhibitory control in the prefrontal cortex when children with obesity see food cues [22, 23], and increased susceptibility to larger portion sizes in overweight adults [24]. Even if the value of a toy is substitutable with the value of food in general, it may be possible that overweight and obese children have a higher value for food than they do for a toy premium. To answer our question whether BMI will moderate or mute the effect of the toy on children’s meal bundle choices, we assessed BMI adjusted for age and sex in all of our participants. If overweight or obese children assign a greater value to larger food portions than they assign to a toy substitute, we would expect our manipulation would not entice these children to choose a smaller-sized meal.

At present, there is little experimental evidence evaluating the potential reinforcing value of toys in smaller-sized meal bundle choice [25]. The goal of the present work was to test the effectiveness of toys in lowering meal bundle choice and consumption. To do so, an experiment was conducted in the field with actual meal bundles (i.e., McDonald’s Happy Meals), across time (i.e., two repeated trials), and while considering age- and sex-adjusted BMI as well as sex, age, and hunger level. Two repeated trials allowed us to test if the effect would change over time. Such a “wear out” out effect could be possible if children become tired of the toy offering, in which case the toy could lose reinforcement value compared to the larger portion size. To control for this possibility, we added the factor *time*.

## Materials and Methods

### Design

The study employed a mixed experimental design with *toy pairing* (toy paired with regular-sized meal bundle; toy paired with smaller-sized meal bundle) as the between-subjects independent variable, *time* (time point T1; time point T2) as the within-subjects independent variable, and *meal choice* as the dependent variable. The research was approved by the University of Arizona’s review board, permitted by teachers, and disclosed to parents in writing. Disclosure form and study information was provided to parents via mail. School principals, teachers, and staff provided consent on behalf of the children enrolled in the study. Each participant made two separate food choices on two different days (T1 and T2, separated by one week for one school and three days for the other) in this repeated-measures experiment. Participants remained in their originally assigned conditions (i.e., toy paired with regular-sized or with smaller-sized meal bundle) between the two time points. The toys differed between T1 and T2.

### Sample

One hundred and nine school children participating in a summer program of two elementary schools in the Tucson, Arizona metropolitan area (55 females; *M*_*age*_ = 8.53 years, *SD*_*age*_ = 2.14; *M*_*BMI*_ = 18.30, *SD*_*BMI*_ = 4.42) participated individually. The schools were randomly selected from the Tucson Unified School District and include students with mean household income of $54,952 [26]. On average, male participants were 8.97 years of age (*SD* = 2.28) and female participants were 8.21 years of age (*SD* = 1.96). The age range in our sample was assigned to us by the school. The children were varied in age because all children at both schools always ate lunch together in the summer program. Our sample is appropriate because the age range of children was that of typical elementary schools [27], and is considered to be critical in healthy development, especially because obese children in this age range are likely to be obese as adults [28]. On average, male BMI adjusted for age and sex was 17.46 (*SD* = 3.15) and female BMI adjusted for age and sex was 19.33 (*SD* = 5.46). BMI varied between 12.50 (1^st^ percentile) and 37.60 (99^th^ percentile) with a mean BMI of 18.30 (*SD*_*BMI*_ = 4.46). There was no significant difference in BMI adjusted for age and sex between the two conditions (*M*_*toy paired with regular-sized meal bundle*_ = 18.40, *SD* = 4.73 vs. *M*_*toy paired with smaller-sized meal bundle*_ = 18.21, *SD* = 4.16), *t*(64) = .17, *P* = .862. We also tested differences in BMI *z*-score and BMI-for-age-percentile. Typically, children at or above the 95^th^ percentile are considered obese and children between the 85^th^ and 95^th^ percentile are considered overweight [29]. In our sample, 14 children were considered to be obese and seven children were considered to be overweight. There were neither significant differences between conditions in BMI *z*-scores (*M*_*toy paired with regular-sized meal bundle*_ = .81, *SD* = 3.00 vs. *M*_*toy paired with smaller-sized meal bundle*_ = .54, *SD* = 1.29, *t*(64) = .48, *P* = .636), nor BMI-for-age-percentile (*M*_*toy paired with regular-sized meal bundle*_ = 57%, *SD* = 32% vs. *M*_*toy paired with smaller-sized meal bundle*_ = 64%, *SD* = 31%, *t*(64) = -.92, *P* = .361). Students who were not present for one day of the study were treated as missing values (in T1 78, and in T2 77, children participated). The participation and attrition rates depended upon current school enrollment and daily attendance in the summer program. Some families did not send their child to the summer program consistently from week to week, which explains the variability in sample size. The results do not differ qualitatively if we focus the analysis only on those children present on both days; as such, we included the entire data in the main analysis even if one out of two data points were missing. Data from the full sample was usable for further analyses.

### Monetary allowance

Usually, students in the summer program are provided free lunch every day. During the days of the experiment, free lunch was not provided. Instead, we provided students with the option to purchase their lunch with an allowance. In order to ensure each student was able to purchase the meal bundle, if they so desired, we provided students with monetary coupons. Several weeks prior to the experiment, parents received monetary coupons worth $6.20 from the experimenter to be given to their child; $3.10 for each day of the experiment, reflecting the average price of a McDonald’s Happy Meal [30]. Parents were told their child could choose to purchase lunch items with the allowance or receive the actual monetary face value. The goal of this approach allowed for children’s psychological incorporation of $6.20 into their wallets prior to experimentation and simulated realistic, incentive-compatible food purchase decisions. To make the logistics of paying for the food easier, children brought their coupons to school only if they wanted to be reimbursed instead of purchasing the meal bundle. However, all participants purchased meal bundles with their allowance and did not request a reimbursement.

### Procedure

Around lunchtime, meal bundles were prepared fresh at a near-by McDonald’s restaurant, picked up by the experimenter, and delivered to the school’s cafeteria. Children entered the cafeteria in a single-file line determined by the teachers. Upon entering the cafeteria, children were randomly assigned one by one from the order they were in the line to sit at one of two tables (the two conditions). The assignment of each child alternated between the two tables on which the experimenter had placed a sheet of paper face down, displaying the choice options and hunger scale, and a pen. Once seated, the experimenter told participants to turn around their choice sheet, look at the two choice options, and without talking to their neighbor mark which meal they would prefer to eat. The assignment of children to different tables depending upon their condition assured that participants would not be able to see choices different form their own. Once they selected their meal, the children in the first condition lined up to accept their food and were taken out of the cafeteria to eat at a location away from the children of the other condition. The children in the second condition followed suit, after the first condition exited. All children present participated.

In the “*toy paired with the regular-sized meal bundle*” condition, participants were offered the choice between a regular-sized Happy Meal, including a toy, and a smaller-sized Happy Meal, *excluding* a toy (this condition was coded 0). The regular-sized Happy Meal included a McDonald’s cheeseburger (290 calories), French fries (110 calories), Strawberry Yoplait^™^ Go-Gurt (50 calories), and chocolate milk (130 calories), totaling 580 calories [31]. The smaller-size meal included a McDonald’s cheeseburger and the chocolate milk, totaling 420 calories. The composition of the smaller-sized meal bundle was altered by removing certain items from the regular-sized meal bundle. The main item, a burger, was identical between the two conditions. We were able to remove 160 calories from the regular-sized meal bundle to create the smaller-sized one. Toys were action figures for males and doll animals for females, and were also bought from McDonald’s together with the meal bundles.

In the “*toy paired with the smaller-sized meal bundle*” condition, participants were offered the choice between a regular-sized Happy Meal, excluding a toy, and a smaller-sized Happy Meal, *including* a toy (this condition was coded 1).

On their choice sheets, participants also responded to an established item that asked how hungry they were (1—*not at all hungry*; 5—*very hungry*) [32]. The experimenter recorded each participant’s first name, sex, and date of birth. Participants were then asked to come forward and claim their choice. Choices of regular-sized meal bundles were coded as 0 and choices of smaller-sized meal bundles were coded as 1. Participants then returned to their tables, consumed the food, and returned to their classrooms. After the children had left, the experimenter took photographs and counted amounts and types of leftover foods (condition-specific). There was no considerable difference in leftovers between conditions. Foods left over in the first condition were 8.25 burgers, .5 fries, 6 yogurts, and 8 milks, and foods left over in the other condition were 10.25 burgers, 0 fries, 5 yogurts, and 7.75 milks. However, it is important to note, that considering the difference between total calories consumed and total calories from the foods leftover, there was a considerable difference between average calories consumed among children who chose regular-sized meal bundles (*M =* 999.5 calories consumed) and those who chose smaller-sized meal bundles (*M =* 233.15 calories consumed). The formula used to arrive at these average calories consumed per meal-size choice was as follows: [(total calories consumed per choice condition *minus* total calories leftover per choice condition) *divided by* number of participants who selected the choice conditions]. Certified school nurses provided available height and weight information for participants (21 measurements from each school were reported missing and were not included in the analyses). The experimenter calculated the body-mass-index (BMI), BMI *z*-score, and the corresponding BMI-for-age-percentile based on the Centers for Disease Control and Prevention’s BMI-for-age growth chart using the standard age- and sex-adjusted calculator for children [29].

## Results

### Positive effect of toy pairing on meal bundle choice

**Fig 1:**
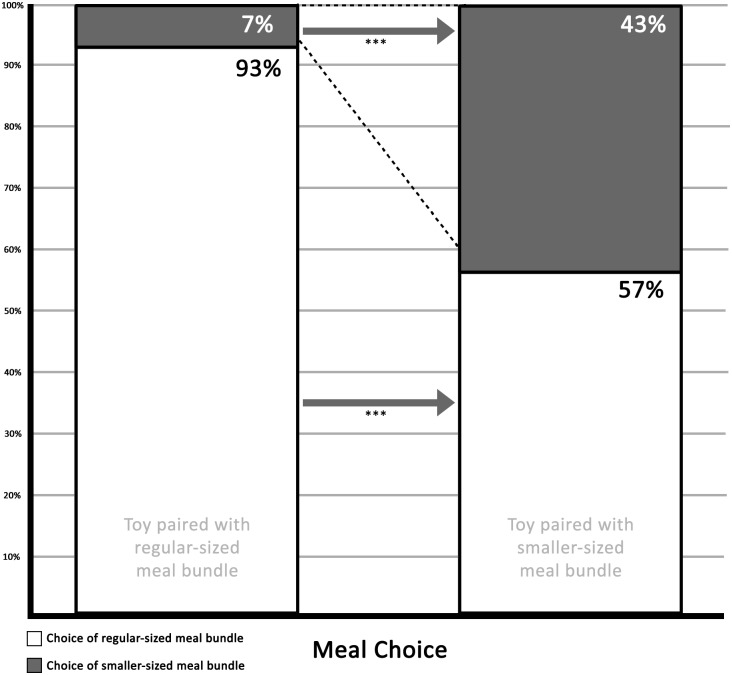
Average choice of meal bundle when paired with toy.

We analyzed the extent to which children would choose smaller-sized meal bundles if paired with a toy. We regressed toy pairing, time, and the interaction term between toy pairing and time on smaller-sized meal bundle choice by estimating a random-intercept logistic regression model with subject as clustering variable and time as time variable [33]. The regression model confirmed a significant positive direct effect of toy pairing on meal choice, *B* = 3.19, *SE* = .97, *z* = 3.28, *P* = .001, 95% CI [1.28; 5.10], showing that a greater proportion of children selected the smaller-sized meal bundle when this meal was paired with a toy. We next analyzed the extent to which the direct effect of toy pairing on meal choice was stable across T1 and T2. Both the direct effect of time (*P* = .501) and the effect of the interaction term (*P* = .171) on meal choice were nonsignificant, suggesting the effect of toy pairing on meal choice was stable over time. At T1, choice of the smaller-sized meal bundle was significantly greater for children in the “toy paired with smaller-sized meal bundle” condition (44% chose the smaller-sized meal bundle) compared to those in the “smaller-sized meal bundle with no toy” condition (only 3% chose the smaller-sized meal bundle), *χ*^*2*^ = 18.86, *P* < .001. At T2, a similar pattern was observed: choice of the smaller-sized meal bundle was significantly greater for children in the “smaller-sized meal bundle with no toy” condition (36% chose the smaller-sized meal bundle) compared to those in the “toy paired with regular-sized meal bundle” condition (only 8% chose the smaller-sized meal bundle), *χ*^*2*^ = 8.52, *P* = .004. We want to clarify that the comparison being made is the choice of the smaller sized meal between conditions. Table 1 summarizes the aforementioned percentages. Fig 1 illustrates the choice percentages averaged over both time points of the *smaller*-sized meal bundle as compared to the choice percentages of the *regular*-sized meal bundle.

**Table 1:** A greater proportion of children selected the smaller-sized meal bundle when this meal was paired with a toy.

	Choices at Time 1		Choices at Time 2	
	Chose regular-sized meal bundle	Chose smaller-sized meal bundle	Chose regular-sized meal bundle	Chose smaller-sized meal bundle
Toy paired with regular-sized meal bundle	97%	3%	92%	8%
Toy paired with smaller-sized meal bundle	56%	44%	64%	36%

### Nonsignificant moderating effects of BMI, BMI *z*-score, and BMI-for-age-percentile

We analyzed the extent to which BMI would affect the direct effect of toy pairing on meal choice. We regressed toy pairing, BMI, and the interaction term between toy pairing and BMI on smaller-sized meal bundle choice by estimating a random-intercept logistic regression model with subject as clustering variable and time as time variable [33]. The regression model confirmed a significant positive direct effect of toy pairing on meal choice, *B* = 2.47, *SE* = 1.07, *z* = 2.31, *P* = .021, 95% CI [.38; 4.55]. Both the direct effect of BMI (*P* = .656) and the effect of the interaction term (*P* = .125) on meal choice were nonsignificant, suggesting the effect of toy pairing on meal choice was neither moderated nor nullified by BMI. We also regressed toy pairing (*B* = 2.70, *SE* = 1.12, *z* = 2.41, *P* = .016, 95% CI [.50; 4.90]), BMI-for-age-percentile (*P* = .718), and the interaction term (*P* = .112) on meal choice, which substantiated the results from the regression model using BMI as independent variable. When the variables sex, age, and hunger level were added to the regression model as controls, the main effect was marginally significant (*P* = .068) but neither the effects of BMI (*P* = .569), sex (*P* = .348), age (*P* = .164), hunger (*P* = .628) nor the interaction term (*P* = .222) were significant.

## Discussion

Despite efforts to introduce “healthier” choices, it is regularly left to restaurants to define “healthy”; for example, replacing an “unhealthy” cookie with a “healthy-looking” yogurt high in sugar. With high childhood overweight and obesity rates, the question may not only be how to improve the nutritional value of foods but also how to stimulate children to choose *less*. In the present research our emphasis is on encouraging children to consume *less* (smaller portions), avoiding the problem of defining “healthy” (e.g., is substituting fat [French fries] with sugar [apple slices with caramel dipping sauce] a “healthier” option?). This approach is not complex but could be effective, as it entails cutting, for example, 160 calories from a children’s meal bundle. Our finding suggests that using the reinforcement value of a toy in children’s meal bundles is a simple and effective strategy for reducing portion size consumed by children. Instead of restricting food options in a way that can lead to resistance and rejection of the smaller-sized bundle option [34, 35], the present research suggests utilizing the reinforcing value of a toy to *incentivize* children to choose less. Importantly, in our research, the identified effect of toy on choice was neither moderated nor nullified by BMI. This result was surprising, given prior insight that food cues are more attention-grabbing in individuals with obesity [22, 23], implying that a toy might matter less to children with higher BMI. Therefore, the non-moderating effect of BMI provides an additional robustness check for the direct effect of toy on smaller-sized portion choice; this finding corroborates research exploring the predisposition to overeat in response to larger portion sizes, regardless of BMI [36]. Indeed, we found—without a toy—children chose larger portions more often (in support of [36]); yet, including a toy moves even overweight and obese children’s choices toward the smaller-sized portion. The effect showed stability across two repeated choices made on two different days separated by one week. Additionally, we found children do not tire of the smaller-sized meal and toy bundle when repeatedly offered. Because fast food restaurants have mechanisms in place to vary the offered toy premium on a frequent basis, we would expect that with regularly changing toy collectibles, children could be kept motivated to choose less food over time. In essence, we utilize the notion of a reinforcement value of a widely-used food-marketing tactic—a toy accompanying a children’s meal bundle—to help children change their current behavior and choose *less*.

### Theoretical contributions

Our work contributes to the literature in a variety of meaningful and practical ways. Given theory that says food and nonfood incentives can represent a “common currency” in the brain [25], our real-choice intervention should be theoretically applicable to a variety of food-choice settings that involve portion size selections. The commensurability of a nonfood incentive such as a toy with the natural reward of eating more tasty calories can help children to exchange a bigger portion of food for a smaller portion of food, if paired with a toy. The theoretical implications of our field experiment suggest that the reinforcement value of a toy could be a particularly strong strategy for improving children’s portion size selections. Before this understanding, the present research followed the advice that people should just “eat less” [37]. Especially for unsupervised children, however, this may be easier said than done. Because it is hard to exercise self-discipline to eat less (especially for children) [38, 39], the present work highlights the potency of nonfood incentives in helping children to choose and consume less food: a small, inexpensive toy premium. Beyond behavioral, psychological and neurological investigations of portion-choice [25], the impact of toy premiums on “healthier” choices [40] and the impact of priming children with a role model on healthier choices [41], our research is the first to exemplifies the impact of a toy on children’s meal bundle choice in a field experiment. Thus, our work contributes to and extends current knowledge of the effect of toy premiums on portion choice in children’s meal bundles.

### Limitations and research opportunities

This research presents limitations that offer opportunity for future research. First, the manipulation could be perceived as a choice between two different meal compositions instead of between “regular-sized” and “smaller-sized.” To keep all else equal, it would be necessary to offer identical foods, one of which is exactly the half portion of the other (e.g., a whole burger and half of a whole burger). To maintain a choice situation as similar to reality as possible, we did not offer a half burger choice because McDonald’s does not currently offer this option. Second, another limitation is that our experimental design includes only two time points. Although the power of a toy has clearly been successful as a food marketing tactic [9, 10], there is still the possibility of a wear out effect over longer periods of time that future research could investigate. Third, based on prior research, the authors assumed the inclusion of a control condition, in which there was no toy offered, was unnecessary because in the absence of an incentive, a larger meal should always be more desirable than a smaller meal [12, 25, 42, 43]. Future research could compare additional conditions (e.g., baseline). Finally, future research could also explore the effect of a premium on older children or adults and also on food types different from fast food.

### Implications

Recent research acknowledges the necessity of additional fast food menu improvements [44], especially because restaurants’ menu changes have only led to modest decreases in energy reduction [34, 45]. In lieu of immediate menu changes, an innovative suggestion for helping consumers to choose less is to use framing strategies that present healthier options as more appealing in comparison to less-healthy options [46]. We provide novel insights into one possible strategy to curb childhood obesity and overweight by utilizing children’s meal bundles to illustrate the reinforcement value of nonfood incentives; the power of the toy. This information is beneficial to consumers, firms, and policy makers interested in manipulating children’s food choices to be both healthy and appealing. Furthermore, the logistics of implementing this strategy in restaurant settings seems to be relatively straightforward and economically efficient. Because fast food restaurants already have toy-meal bundles on their menus, implementation of a new bundle of smaller-sized meals with a toy (while omitting the toy from the regular-sized meal bundle) is likely to be operationally feasible. More importantly for restaurant businesses, the question is whether it is also economically reasonable for restaurants to make such changes. In our study, we gave a monetary allowance to all participants prior to the experiment to simulate children having to pay for their choice. Meal bundle variations were priced equally ($3.10). All participants opted to purchase a meal bundle and none claimed to keep their monetary allowance. Although this finding could be replicated in an adult sample in which participants have to actually pay with their own earned money, our finding provides some preliminary evidence that consumers may actually be willing to pay similar dollar amounts for the bundle of less food and nonfood incentive than for the regular-sized food portion alone.
